# EUV photofragmentation study of hybrid nonchemically amplified resists containing antimony as an absorption enhancer†

**DOI:** 10.1039/c7ra12934c

**Published:** 2018-03-19

**Authors:** Cleverson Alves da Silva Moura, Guilherme Kretzmann Belmonte, Pulikanti Guruprasad Reddy, Kenneth E. Gonslaves, Daniel Eduardo Weibel

**Affiliations:** Department of Chemical Physics, Chemical Institute, UFRGS Porto Alegre 91501-970 RS Brazil danielw@iq.ufrgs.br; School of Basic Sciences, Indian Institute of Technology Mandi Mandi – 175001 Himachal Pradesh India

## Abstract

A detailed investigation to understand the mechanism of the resist action at a fundamental level is essential for future Extreme Ultraviolet Lithography (EUVL) resists. The photodynamics study of a newly developed hybrid nonchemically amplified 2.15%-MAPDSA–MAPDST resist using synchrotron radiation excitation at 103.5 eV (12 nm) is presented. Antimony was incorporated in the resist as a heavy metal absorption center in the form of antimonate (2.15%). The results showed the fast decomposition rate of the radiation sensitive sulfonium triflate. HR-XPS and sulfur L-NEXAFS spectra of the copolymer films revealed that after irradiation the Ar–S^+^–(CH_3_)_2_ sulfonium group bonded to the phenyl ring resisted the EUV excitation. Those results confirmed the polarity switching mechanism from hydrophilic sulfonium triflates to hydrophobic aromatic sulfides obtained in previous results. The inorganic component SbF_6_^−^ included in the resist formulations as an EUV absorption enhancer was particularly illustrative of the photofragmentation process. F 1s and O 1s HR-XPS spectra showed that fluorine remains linked to the antimony, even after 15 min of irradiation. A change of the antimony oxidation state was also observed with an increase in irradiation time. The presence of the heavy metal may control the high energy deposited on the resist which finally led to very well resolved 20 nm isolated line patterns by EUVL. The 10 times improved sensitivity compared with previous poly-MAPDST resists studied in the past showed the potential of this class of hybrid resists for next generation semiconductor industry applications.

## Abstract 2

Understanding the EUV photofragmentation mechanism in a highly sensitive hybrid n-CAR containing SbF_6_^−^ as an absorption enhancer.

## Graphical abstract

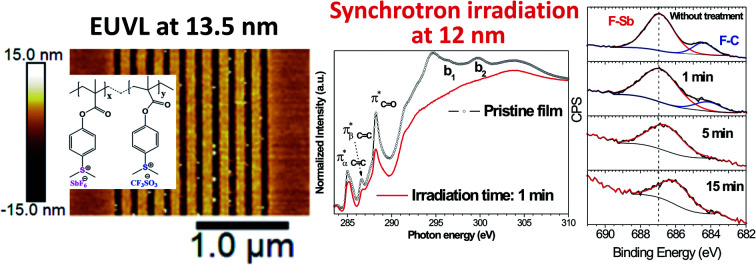

## 1 Introduction

As the potential of extreme ultraviolet lithography (EUVL) is extended beyond the 10 nm range and below,^1^ it is clear that a shift in resist design is paramount.^2–4^ The concepts to be considered, among others, include essentially non-chemically amplified resists (n-CARs) and hybrids.^4–15^ The design paradigm has to incorporate the basic principles of conventional resists superimposed with the specific requirements of EUVL for attaining the lower nodes.^12,15,16^ The interaction of the resist thin films with high energy EUV photons (13.5 nm) is a very complex process triggered by EUV radiation that breaks the chemical bonds and simultaneously produces ablation and a high yield of secondary electrons.^15,16^ This results in degassing which causes chemical and morphological changes in the resist surface. One of the key challenges in EUVL is simultaneously meeting resist performance targets like sensitivity, resolution, etch resistance, and line edge roughness (LER).^15^ Therefore, photons in EUVL produce unique challenges that need detailed investigation *via* a proper light source and complementary *in situ* sensitive analytical techniques.

A recent review on electron beam lithography summarizes the new developments in resists and classifies them according to their various functions, merits and chemical compositions.^17^ Actual polymer films for next generation lithography have poor EUV absorption cross sections; low etch resistance and low performance for high resolution patterning. In this sense, organic/inorganic hybrid photoresists have received attention in recent years due to their combined functionalities arising from both inorganic and organic components. It has been shown that the incorporation of inorganic units such as metals or metal nanoparticles (NPs) give to the organic photoresists a higher etch resistance with simultaneous increase of the absorption cross section in the EUV region.^4,5^ For example, new designs for EUV resists based on organometallic carboxylates containing antimony, bismuth, tin or tellurium were prepared with the objective to use the high EUV optical density of the metals to increase the photon absorbance of thin films.^18^ The results showed that the resists containing metals had higher sensitivity compared with only organic ones, being the most sensitive the resist with antimony and tellurium the less. Incorporation of NPs in hybrid photoresist materials has also been investigated with the objective to obtain high absorption centres for EUVL.^13,18^

Attempts to understand the mechanisms after the absorption of the high energy EUV photons were also carried out in resists containing metals and NPs.^11,19,20^ In an interesting photolithographic study of the properties of tin-oxo clusters, the effect of resist sensitivity of the structures of the carboxylic counter-anions and organic ligands was investigated.^11^ The authors hypothesized that an important mechanism of carbon–tin bond homolysis during exposure was responsible for the high resolution capabilities of those materials. They proposed that the higher optical densities of tin and oxygen atoms compared to carbon atoms provided superior EUVL performance based on more efficient utilization of the EUV photons. A mechanism investigation on Hf-based hybrid photoresists have been performed by studying the influence of surface organic ligands on the physicochemical properties of the hybrids resists.^19^ In spite, the authors studied the NPs size dependence with the ultraviolet (UV) irradiation time; they were able to correlate the UV data with the EUVL pattering results. They showed a relationship between the very high sensitivity to EUV radiation with the NPs size that finally led to high sensitivity and high resolution patterns.

In the last years we have studied the photofragmentation of several n-CAR homopolymers and co-polymers under EUV synchrotron radiation (SR) excitation.^4,9,15,16,21^ The obtained results showed that the photodegradation processes affected mainly the triflate group but also the carbon backbone of the resists. In those works it was hypothesized that the neutral sulfide Ar–S–CH_3_ is formed after irradiation rendering the irradiated area insoluble in the developer. It was found a direct effect of the EUV irradiation changing the resist polarity from initially hydrophilic to hydrophobic. Recently, the concepts of hybrid resists incorporating EUV absorbing metals such as antimony in a n-CAR platform and the EUV lithography results have also been studied.^22,23^ The n-CAR 1.5 and 2.15% MAPDSA–MAPDST (where MAPDST = (4-(methacryloyloxy) phenyl)dimethylsulfoniumtriflate and MAPDSA = (4-(methacryloyloxy)phenyl)dimethylsulfoniumhexafluoroantimonate) resists revealed improved sensitivity as compared to the poly-MAPDST resist for EUVL in spite the low concentration of hexafluoroantimonate used. The sizing dose used for high resolution line patterns when the SbF_6_^−^ units were incorporated in the 1.5% and 2.15% resists resulted in the improvement of the resists' sensitivity by 2.5 and 10 times, respectively, compared to the poly-MAPDST resist.^15,21,23^

Therefore, herein it is presented the photofragmentation investigation of hybrid 2.15% MAPDSA–MAPDST resist incorporating EUV absorbing metals such as antimony. The resist structure incorporates a radiation sensitive sulfonium triflate and the inorganic moiety SbF_6_^−^ acting as a sensitivity enhancer for 13.5 nm photons. The 2.15% MAPDSA–MAPDST resist was chosen for the present study because of its improved lithography performances under EUVL. Evidence from our previous research findings,^24^ showed that the 2.15%-MAPDSA–MAPDST resist has high EUV sensitivity and resolution for ∼20 nm line features than 1.5%-MAPDSA–MAPDST resist. This effect was mainly due to the presence of high inorganic hexafluoroantimonate content in the 2.15%-MAPDSA–MAPDST resist backbone. The results showed that the presence of SbF_6_^−^, even in low concentration, led to a higher etch resistance while maintaining the required processing properties of the resists. This study was carried out using SR as highly monochromatic photon excitation source at 103.5 eV (12 nm). Near-edge X-ray absorption fine structure (NEXAFS) and X-ray photoelectron spectroscopy (XPS) were used as highly sensitive surface analytical techniques for characterization to follow the surface chemical changes after EUV irradiation.

## 2 Experimental section

### 2.1 Materials

**Fig. 1:**
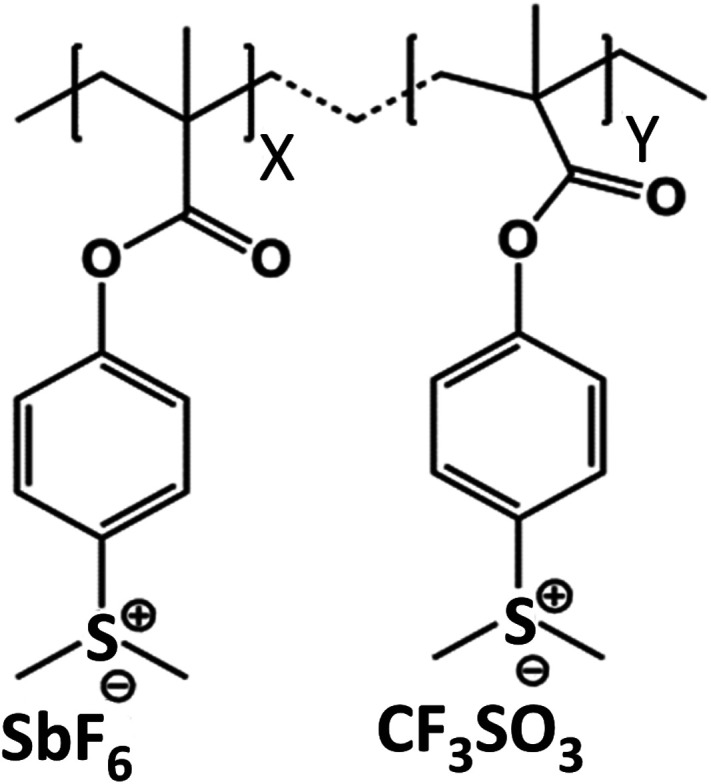
Chemical structure of the 2.15% MAPDSA–MAPDST resist.

The 2.15%-MAPDSA–MAPDST resist (see Fig. 1) was synthesized according to previous protocols by the co-polymerization between the starting organic monomer, MAPDST, and the hybrid monomer, MAPDSA, using azobisisobutyronitrile (AIBN) as a free radical initiator.^23^ Oxygen (99.999%) and argon (99.999%) were obtained from White Martins-Praxair, Inc. and used as received. They were used for surface oxidation during the experiments and for thin film preparation, respectively. Potassium trifluoromethanesulfonate (98%) was purchased from Sigma Aldrich and was also used as received.

### 2.2 Thin film preparation and EUV exposure details

The resist solution (3 wt%) was prepared by dissolving 2.15%-MAPDSA–MAPDST resist in acetonitrile. The smooth thin films (∼45 nm thickness) were achieved by spin coating the above resist solutions at 4500 rpm for 60 s on 4′′, p-type HMDS coated Si wafers. The EUV exposure of these thin films was performed at LBNL using an ALS MET Standard Mask IMO228775 with field R4C3 (LBNL low flare bright-field). The negative patterns were generated by developing of the EUV exposed Si substrates in an aqueous 0.022 N tetramethylammonium hydroxide (TMAH) developer for 15 s. Field Emission Scanning Electron Microscope (FE-SEM- Carl Zeiss, Ultra Plus) and Atomic Force Microscopy (AFM – Dimension Icon from Bruker) were utilized for investigating the critical dimensions of the line and other complex patterns obtained.

### 2.3 Synchrotron radiation studies

The photofragmentation study of the 2.15% MAPDSA–MAPDST resist was carried out following the methodology already used in previous works.^21^ Briefly, the 2.15% MAPDSA–MAPDST resist films were prepared using the spin coating technique from a 10^−4^ mol L^−1^ acetonitrile solution on Si wafers of about 5 × 10 mm in size. The thin films were prepared inside a glove box in an argon atmosphere and without the presence of UV light. Irradiation of the resist thin films at 103.5 eV was carried out for a fixed period of time (1, 5 and 15 min) with a spot size of the photon beam at the sample of about 500 μm. Light from 103.5 eV (12 nm) was chosen due to it's high intensity and because it was very close to the actual 13.5 nm used for next generation EUVL. Each irradiation was carried out on pristine films to allow comparison with non irradiated data. The photon intensity at 103.5 eV was about a few hundred times higher than the regions used for the acquisition of the NEXAFS and XPS spectra. Therefore beam damage from XPS and NEXAFS can be ruled out. After irradiation, the samples were transferred to the UHV preparation chamber and pure oxygen at a pressure of about 10^−5^ mbar was introduced for 30 min to neutralize the remaining radicals on the film surface. After oxidation the samples were introduced again into the UHV analysis chamber. The sample position was computer-controlled by an XYZ sample manipulator, which was housed in the UHV chamber (*P* ∼ 1 × 10^−9^ mbar). The right positions of the irradiated areas were easily confirmed by moving ∼1–1.5 mm up–down or right–left the XYZ sample manipulator. Pristine NEXAFS and XPS spectra of the films were recorded outside the irradiated areas.

Potassium trifluoromethanesulfonate thin films, used as a reference of the triflate functional group of the resist, were obtained by dissolving a few mg in methanol at a concentration of 10^−4^ mol L^−1^. The films were formed by spin-coating a drop on Si(100) wafers of about 5 × 10 mm in size.

Synchrotron radiation (SR) experiments were carried out at the Brazilian Synchrotron Light Source (LNLS), Campinas, Brazil. SR monochromatic photons in the range 100 to 1500 eV were obtained using the planar grating monochromator (PGM) beam line for EUV, VUV, and soft X-ray spectroscopy. With a resolving power (*E*/Δ*E*) of 1000–25 000 and a photon flux at the sample between 10^11^ and 10^13^ (photon per s). The Si wafers were directly attached to the sample holder using conducting double-sided tape. Samples outside the UHV chamber were always manipulated in an inert atmosphere and UV light exposure was avoided.

Resist thin films were characterized before and after irradiation using NEXAFS and XPS spectroscopy. NEXAFS spectra were obtained by measuring the total electron yield (electron current at the sample) simultaneously with a photon flux monitor (Au grid). The final data was normalized by the flux spectrum to correct for fluctuations in beam intensity. The software package ATHENA, used for the analysis of X-ray absorption spectroscopy, was used for final treatment of the data.^24^ XPS spectra were obtained using a high-performance hemispheric SPECSLAB II energy analyzer (Phoibos-Hs 3500 150 analyzer, SPECS, Berlin, Germany). The signal of the Au 4f_7/2,5/2_ electrons was used for calibration of the analyzer. The photon energy was fixed at 728 eV for recording the survey and high-resolution (HR)-XPS spectra of F 1s and O 1s. For HR-XPS spectra of C 1s and S 2p the excitation energy was set at 350 eV. A pass energy of 30 eV was used for the survey spectra, whereas HR-XPS spectra of single core atom excitations were recorded with a pass energy of 10 eV. The position of the C–C/C–H signals (C 1s, 285.0 eV) was used for energy calibration. The HR-XPS envelopes were analyzed and peak-fitted after subtraction of the Shirley background, using Gaussian–Lorentzian peak shapes obtained from the CasaXPS software package. Due to overlapping of O 1s and Sb 3p_5/2_, synthetic components from O 1s HR-XPS spectra were combined with survey mode data (TAGS quantification).

## 3 Results and discussion

### 3.1 Untreated resist thin films characterization

**Fig. 2:**
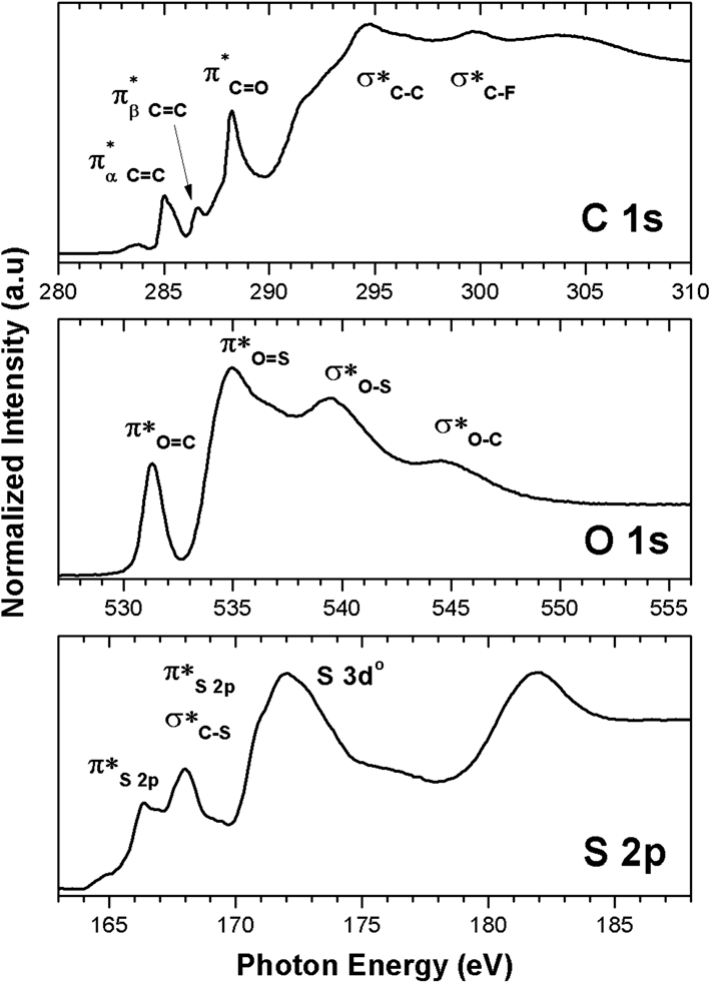
K and L-Near Edge X-ray Absorption Fine Structure (NEXAFS) spectra of the untreated 2.15% MAPDSA–MAPDST pristine resists.

NEXAFS spectra of the carbon K-edge, oxygen K-edge, and sulfur L-edge of the 2.15% MAPDSA–MAPDST pristine resists before irradiation are shown in Fig. 2. The main features in the C 1s absorption spectra (Fig. 2) can be attributed as follows:^25,26^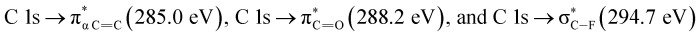 which overlaps with the wide signal at about 292 eV, which can be assigned to a typical 
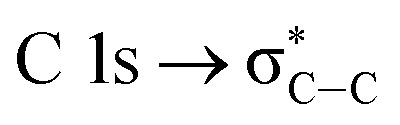
 transition.^25,27^ The second π transition labeled as 
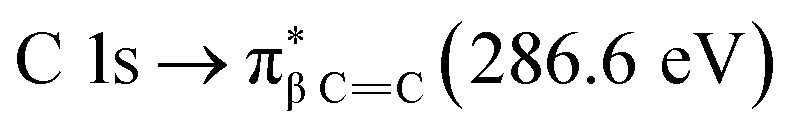
 may be caused by two effects. The first would be an unfolding of the energy levels of C (1s) due to the presence of two different carbon bonds linked to O and S atoms. These different bonds increase the energy of the degenerate states of the π* molecular orbitals of the benzyl ring. The second cause would be the transitions to different π* orbitals.^28^ However, it is necessary to point out that the 
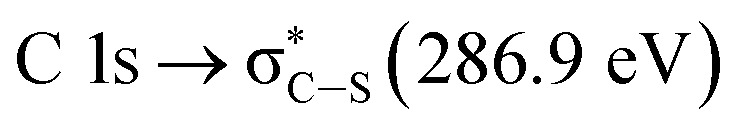
 transition is very well characterized in previous works^29,30^ and may overlap with the 
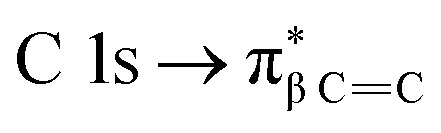
 transition. The weak signal observed at 283.6 eV can be a result of the normalization and data treatment of the data because it was not observed any dependence on the irradiation time (see Section 3.2, carbon K-edge NEXAFS spectra).

The chemical structure of the 2.15% MAPDSA–MAPDST resist shows two types of oxygen sites (see Fig. 1): carbonyl and sulfonated oxygen. The O 1s NEXAFS spectrum (Fig. 2) has a simpler interpretation than the C 1s transitions, which reflect the chemical structure of the resist. The lowest energy signal can be attributed to 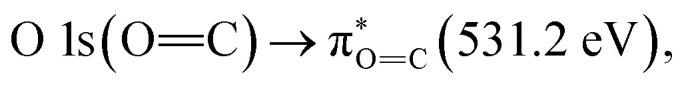 and the second discrete transition may involve the 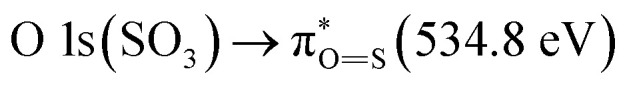 transition.^31^ Finally, the higher energy peaks, at about 540 and 545 eV, can be assigned to 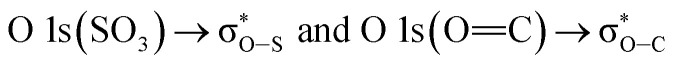 transitions, respectively.^31^

Finally, the sulfur L-edge in Fig. 2 showed an interesting structure: the signal at 166.4 eV can be assigned to electronic transitions involving the spin–orbit split of the S 2p excited species (2p_1/2_ and 2p_3/2_ levels) mainly due to the unoccupied π* antibonding orbitals.^32–34^ A mixture of several transitions can be invoked in the signal that appeared at 168 eV: 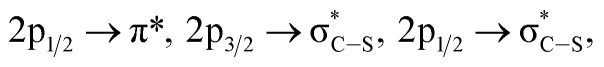 and S 2p → empty S 3d states^33,34^. Finally the signals at about 172 eV and 182 eV may also be assigned to higher energy transitions S 2p → empty S 3d and 
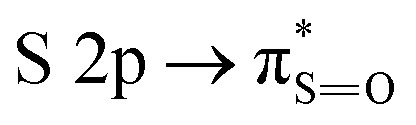
 sulfonic acid functionalities.

**Fig. 3:**
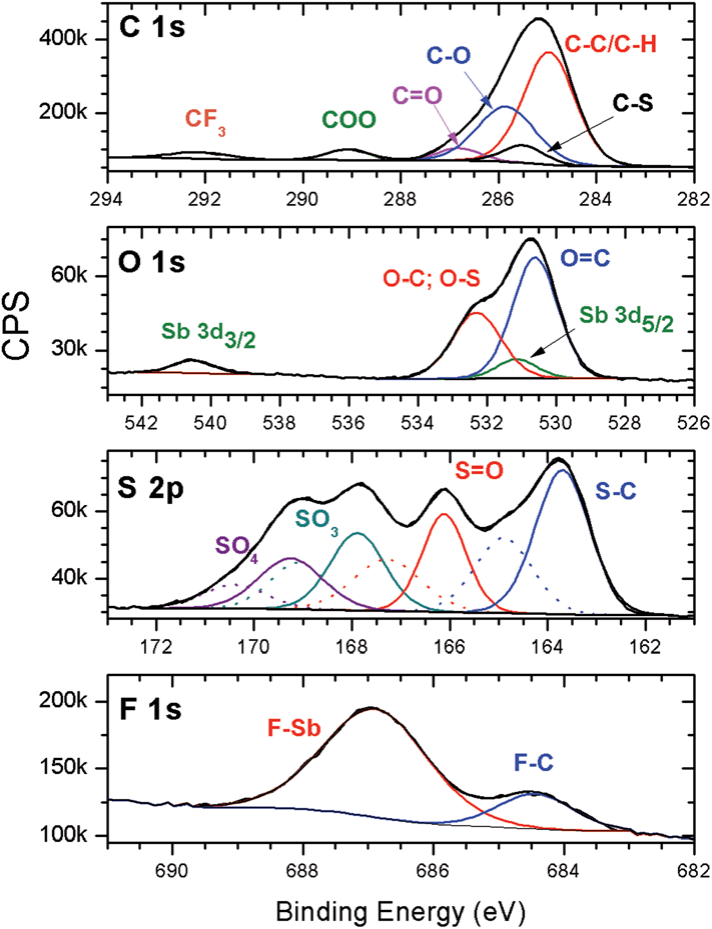
High-resolution XPS spectra of the C 1s, O 1s, S 2p and F 1s envelopes of the 2.15% MAPDSA–MAPDST resist films before irradiation.

High-resolution (HR) XPS data from the C 1s, O 1s, S 2p and F 1s elements of pristine resists were obtained, and the results are shown in Fig. 3. The C 1s envelope of the HR-XPS spectrum of untreated films (Fig. 3) revealed typical signals corresponding to the aliphatic and aromatic contributions (C–C/C–H), C–O, C–S, COO, and CF_3_ functionalities, which agree with the data previously obtained for the MAPDST homopolymer and MAPDST–MMA copolymers.^15,21,35–37^ The S 2p spectrum of the untreated 2.15% MAPDSA–MAPDST surface shows four spin–orbit split doublets, having binding energies that are characteristic of S–C,^38,39^ S=O, SO_3_, and SO_4_.^40^ The presence of four contributions in the S 2p envelope was necessary to fit the experimental signal, which probably could be caused by a possible partial oxidation/degradation of the resists. The relative contribution of this highly oxidized sulfur species was lower than 12%. O 1s and F 1s signals show clear evidence of the presence of Sb. The envelope of the O 1s revels the presence of O=C, the overlapping of O–C and, O–S signals and the presence of the Sb 3d_5/2_ signal.^41–44^ The Sb 3d_5/2_ signal is evident due to the presence of the Sb 3d_3/2_ signal at about 540.5 eV. This peak is situated at about 9.4 eV from the Sb 3d_5/2_ signal, agreeing with the 9.34 eV theoretical value.^44^ Finally, the F 1s HR-XPS spectra shows the presence of two peaks that can be assigned to F–Sb and F–C.^41,42,44^

### 3.2 Photofragmentation study at 103.5 eV

**Fig. 4:**
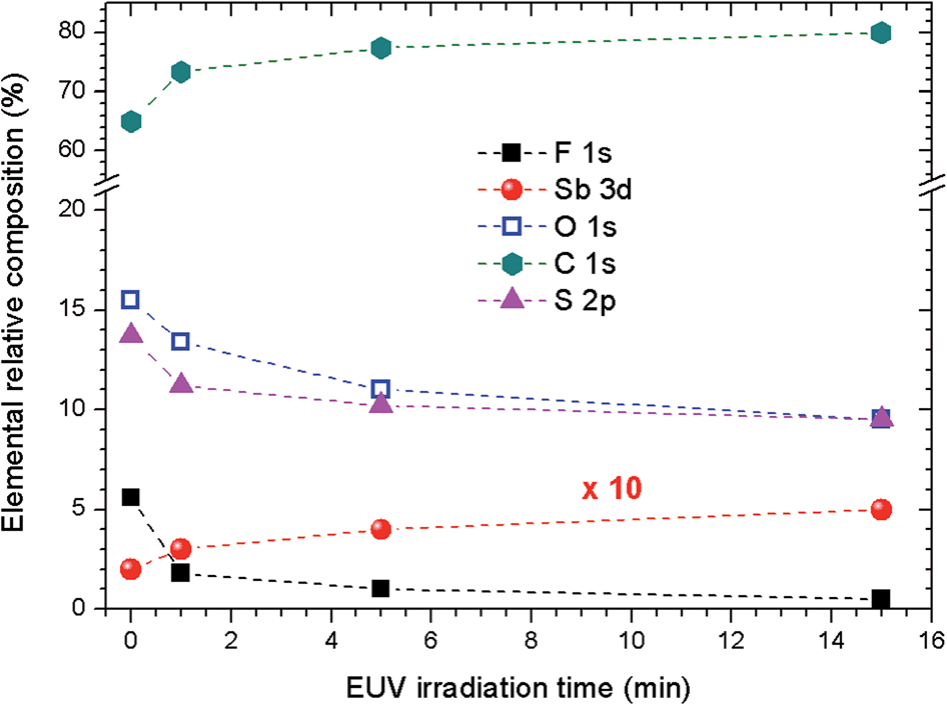
Dependence of the elemental relative atomic percent concentrations of untreated and irradiated 2.15% MAPDSA–MAPDST resist films on the irradiation time. Excitation energy: 103.5 eV. Data obtained from survey XPS spectra. The Sb relative concentration was multiplied by 10 for better presentation.

Survey XPS spectra were obtained from pristine 2.15% MAPDSA–MAPDST resists after irradiation at 103.5 eV for several times intervals (see ESI†). Fig. 4 shows a fast defluorination and a loss of sulfonated groups as a result of an increase in the irradiation time for the copolymer resist films. The results obtained were not surprising as the MAPDST homopolymer thin films have previously shown an efficient desorption process of CF_3_^+^, SO^+^, and SO_2_^+^ fragments when irradiated at 103.5 eV.^15^ Irradiation led to a change in surface chemical composition, with an increase in carbon content and a strong decrease in polar functional groups. Interestingly, the relative concentration of antimony at the surface increased with the increase in irradiation time. The last result will be addressed later on.

**Fig. 5:**
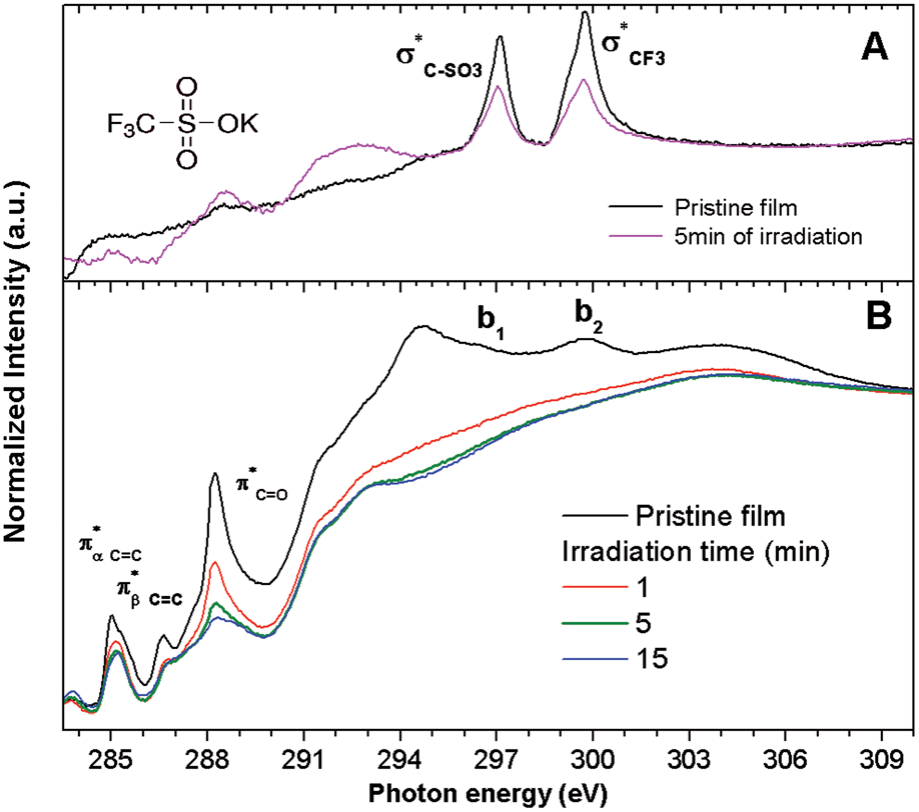
Carbon K-edge NEXAFS spectra of: (A) untreated and SR irradiated potassium trifluoromethanesulfonate thin films. Irradiation time: 5 min (B) untreated and SR irradiated 2.15% MAPDSA–MAPDST resist films for 1, 5 and 15 min of irradiation time. Excitation energy: 103.5 eV.

To obtain more information about the effect of the EUV irradiation on the films, NEXAFS spectra were acquired for irradiated 2.15% MAPDSA–MAPDST resists films. The effect of the 103.5 eV photons on potassium trifluoromethanesulfonate thin films spin-coated on Si(100) was also investigated. This data was used as a reference to better understand the role of the triflate functional group during the photofragmentation process. The triflate results are shown in Fig. 5A. The NEXAFS spectrum of a potassium trifluoromethanesulfonate thin film shows two main signals at 296.4 eV and 299.7 eV in agreement with previous works.^45,46^ These transitions can be assigned to electronic excitation from the C 1s to 
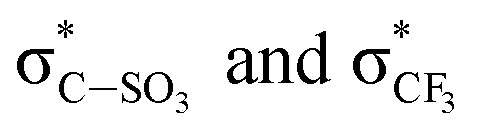
 empty states, respectively. Irradiation of the triflate thin film at 103.5 eV for 5 min led to a strong decrease in the signal intensity of both transitions showing the high sensitivity of the triflate functional group to EUV photons.

The carbon K-edge NEXAFS spectrum of untreated 2.15% MAPDSA–MAPDST resist films shows two signals, identified as b_1_ and b_2_ in Fig. 5B, which may correspond to the triflate functional group of the copolymer. Both of these signals completely disappeared after only 1 min of irradiation at 103.5 eV, proving also the high sensitivity of the triflate group under EUV irradiation when it is incorporated in the resist. A strong decrease in the intensity signal corresponding to a transition 
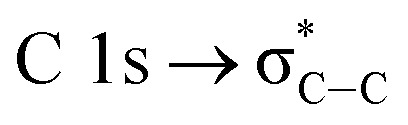
 is also observed (compare with Fig. 2). Photons at 103.5 eV are not resonant and are absorbed by any chemical bond and functional groups of the copolymer. Fig. 5B also shows that the intensity signal of the 
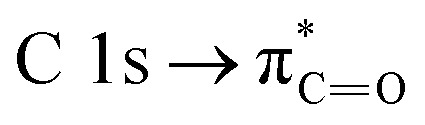
 transition strongly decreased and became slightly wider at longer irradiation times at 103.5 eV, indicating the presence of different chemical C=O groups formed after irradiation/oxidation. 
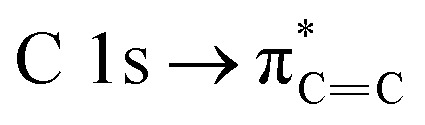
 transitions are also affected to different degrees when the irradiation time increased. The above results were also confirmed using XPS (see ESI†).

**Fig. 6:**
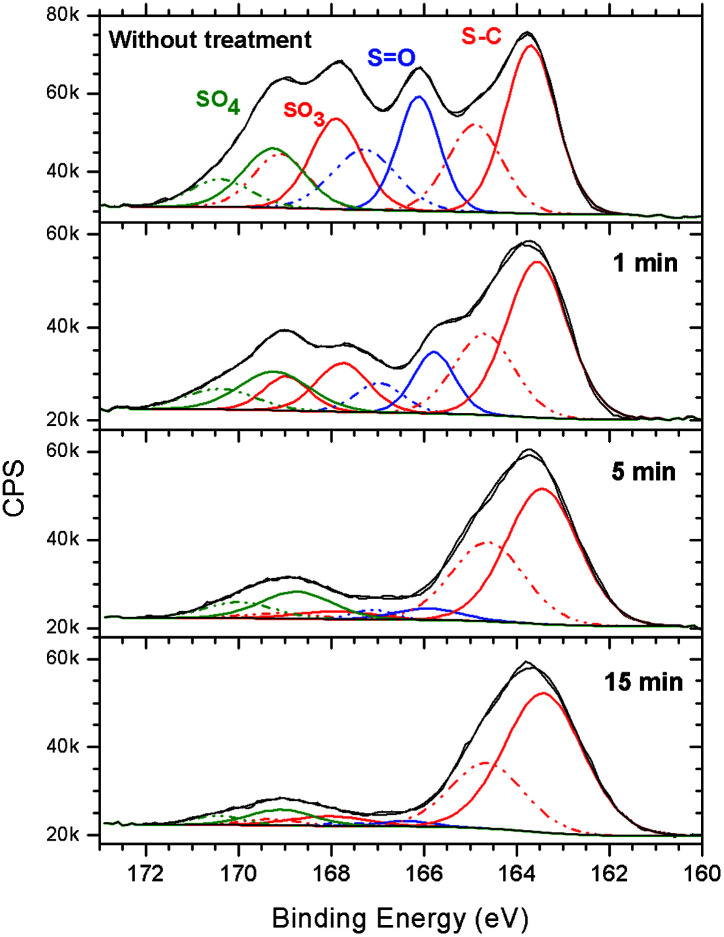
High-resolution XPS spectra of the S 2p envelope of the 2.15% MAPDSA–MAPDST resist films before and after 1, 5, and 15 min of irradiation by synchrotron radiation at 103.5 eV.

**Fig. 7:**
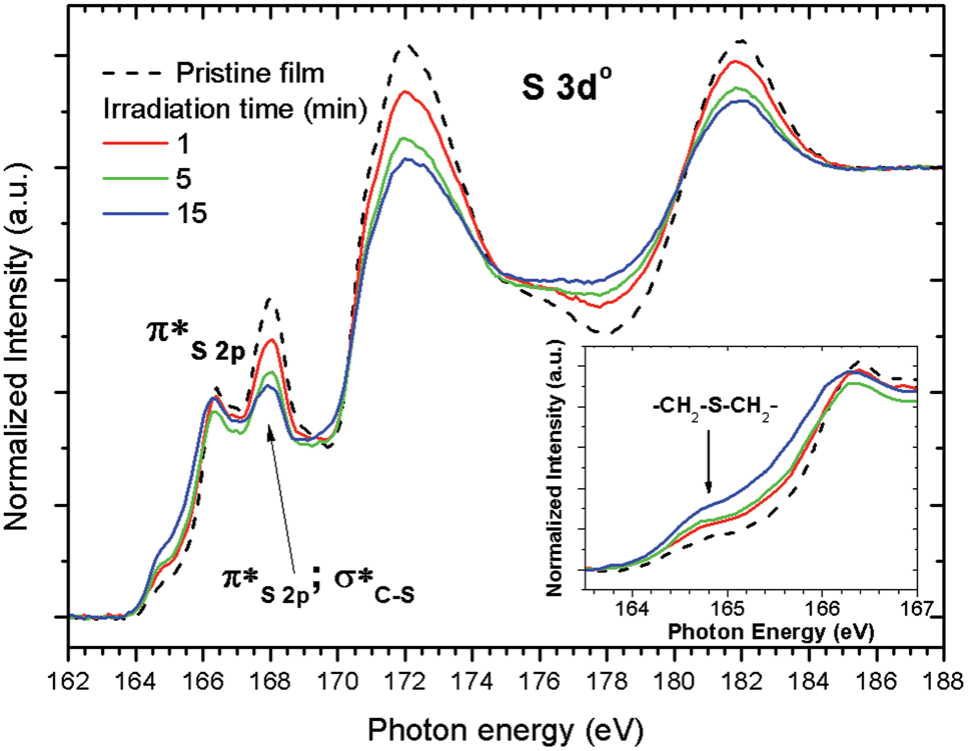
Sulfur L-NEXAFS spectra of the untreated 2.15% MAPDSA–MAPDST pristine resist before and after irradiation at 103.5 eV. The untreated spectrum shown in Fig. 1 is included for better comparison of the data.

HR-XPS spectra of the S 2p envelope showed that when the irradiation time increased, the 2p_3/2_ and 2p_1/2_ signals of the S–C functionality resisted the irradiation (see Fig. 6). A typical HR-XPS spectrum of S 2p (see top of Fig. 6) lost the oxygenated components when the irradiation time increased. After 15 min of irradiation at 103.5 eV, approximately 85% of the HR-XPS S 2p signal corresponded to the S–C functional group. Similar results were already observed for the MAPDST homopolymer resist in previous studies.^15^ In that study, it was assumed that after irradiation, the S–C bonding, probably belonging to the (dimethylthio)phenyl group, resisted the effect of irradiation at 103.5 eV. Due to the photofragmentation process, the irradiated area became insoluble in the developer, changing the polarity from being initially hydrophilic to hydrophobic. Trying to obtain more information of this surface conversion process, sulfur L-NEXAFS spectra were obtained before and after treatment at 103.5 eV. The results can be seen in Fig. 7.

The results presented in Fig. 7 match the information obtained using XPS, *i.e.*, a general loss of sulfonated groups is observed with the increase in irradiation time. However, the higher surface sensitivity of NEXAFS shows that a signal at about 164.8 eV is the only signal that is continuously increasing with the increase in irradiation time (see inset in Fig. 7). In previous HR L-NEXAFS studies of several inorganic and organic sulfur model compounds the authors have used the models spectral as finger prints to identify several organic functional groups in untreated coal.^47,48^ Those functionalities included alkyl and aryl sulfides, alkyl and aryl disulfides, and heterocyclic sulfurs.^47^ The signal that merges (see inset in Fig. 7), when the irradiation time increases may be assigned to a –CH_2_–S–CH_2_– functional group, *i.e.*, a R–S^+^–(CH_3_)_2_ sulfonium group bonded to the phenyl ring in the case of the 2.15% MAPDSA–MAPDST pristine resist. The NEXAFS results of Fig. 7 give more information about the assumed mechanism of polarity change that makes the exposed area less polar than the unexposed area, which in turn leads to differences in solubility of these exposed and unexposed areas.

**Fig. 8:**
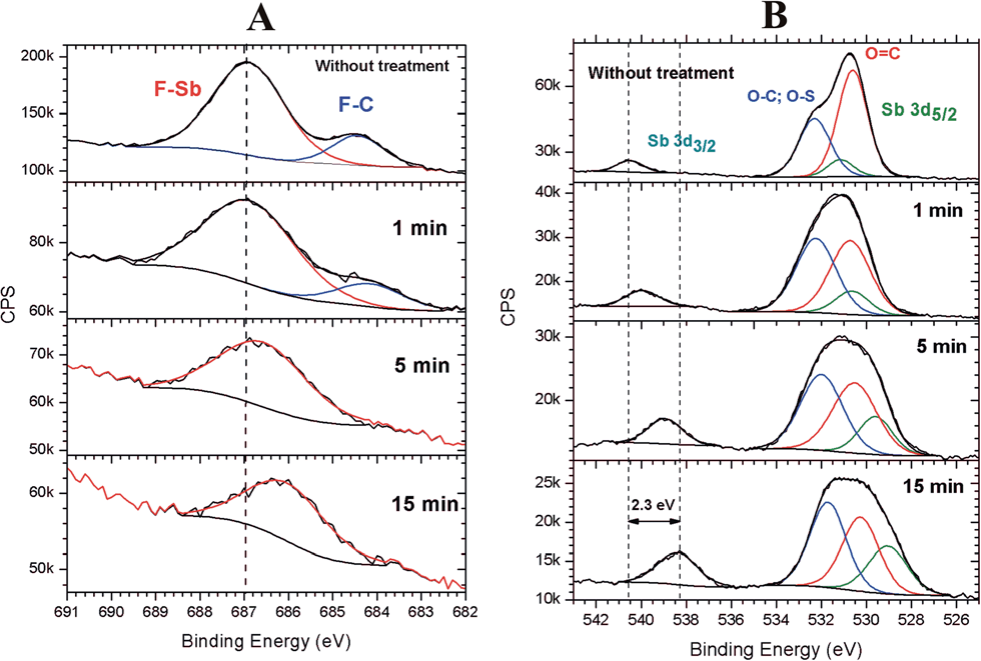
High-resolution XPS spectra of the F 1s (A) and O 1s (B) signals of the 2.15% MAPDSA–MAPDST resist films before and after 1, 5, and 15 min of irradiation by synchrotron radiation at 103.5 eV.

It was observed that the relative concentration of antimony at the surface increased with the increase in irradiation time (see Fig. 4). To gain insight into the photofragmentation process that occurs after the absorption of the highly energetic 103.5 eV photons, HR-XPS data was acquired for the F 1s and O 1s signals. The results presented in Fig. 8A show the evolution of the F 1s signal with the increase in the irradiation time at 103.5 eV of excitation energy. Two signals can be identified in Fig. 8A corresponding to the different chemical environments of the fluorine atoms that can be assigned to the triflate and antimony groups.^36,37,44,49^ It is possible to see in Fig. 8A that the F–C signal originated from the triflate group is strongly affected by the EUV photons and disappeared after 1 min of irradiation. This result agrees with the results shown in Fig. 5 and 6 where the triflate group is easily fragmented after 103.5 eV of photon excitation. However, the F–Sb component in the F 1s signal still remains after 15 min of irradiation. A shift of about 0.88 eV to lower binding energies is also observed indicating a decrease in the electronegativity around the fluorine atoms attached to the antimony atom (see Fig. 8A). A recent study of 1-alkyl-3-methylimidazolium hexafluoroantimonate(v) ionic liquids showed that continuous irradiation with X-ray from an Al Kα source led to a photoreduction of Sb(v) to Sb(iii).^49^ The authors suggested that the X-ray exposure even at room temperatures led to SbF_3_ as a product of photoreduction The SbF_6_^−^ anion. Consequently, the shift of about 0.88 eV shown in Fig. 8A can be assigned to a partial desorption of fluorine atoms from the SbF_6_^−^ anion. The inorganic moiety SbF_6_^−^ has evidently higher resistance to the 103.5 eV photons than the triflate group, even after 15 min of irradiation.

Additional information on the photofragmentation mechanism can be revealed when the O 1s HR-XPS signal is studied as a function of the irradiation time. Fig. 8B shows the HR-XPS spectra in the energy region of O 1s signal and its dependence on the irradiation time. In this binding energy region, it is possible to follow the evolution of a Sb 3d_3/2_ signal independently to the changes observed for the O 1s signal, which in turn overlaps with the Sb 3d_5/2_ signal. As can be seen in Fig. 8B, a continuous shift to lower binding energies is observed in the Sb 3d_3/2_ signal with the increase in irradiation time. That decrease in binding energy was previously observed in preliminary studies of different antimony compounds.^41,49,50^ For example, the shift between the binding energies of NaSbF_6_ and Sb_2_O_3_ was about 2.6 eV to lower energies, which was close to the maximum observed shift of 2.3 eV obtained here (see Fig. 8B). Simultaneously, the chemical composition of the O 1s envelope changed according to the increase in irradiation time. The Sb 3d_5/2_ relative signal composition compared to O 1s signal, increased from 3% in the pristine film to about 10% when the film was irradiated for 15 min. The combination of these results with Fig. 8A may indicate that the antimony remain partially fluorinated in the surface region after irradiation because the F 1s (F–Sb) signal remained after 15 min of irradiation. The above results may show an important role of the inorganic SbF_6_^−^ moiety during irradiation: the SbF_6_^−^ group can function as a component in the composition of the 2.15% MAPDSA–MAPDST resist film that has higher resistant to irradiation compared with, for example, the sulfonium triflate group. The mechanistic origin of the lower rate of fluorine loss under 103.5 eV is an open question. A heavy metal, such as antimony should absorb more EUV photons than lighter atoms. From the results presented here the high rate of EUV photons did not led to a rapid defluorination of the SbF_6_^−^ moiety. The fluoresce properties of antimony compounds in inorganic and organic compounds have been studied in the past.^51–53^ As long as Sb is present and keeps absorbing photons, it will probably emit photons too. Fluorescence measurements were not carried out in the present study. The higher optical density of Sb is possibly contributing to the enhanced sensitivity of the resist (8–10 relative to the carbon optical density of 0–2)^54^ allowing a control of the etching. As it was proposed in a previous work containing tin,^11^ the Sb–F bond homolysis and the higher optical densities of antimony compared to carbon atoms led to a superior EUVL performance by efficient utilization of the EUV photons.

### 3.3 Extreme ultraviolet lithography (EUVL)

**Fig. 9:**
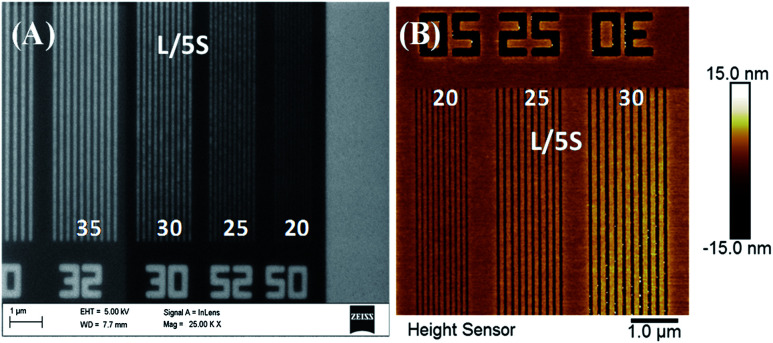
EUV exposed patterns of 2.15%-MAPDSA–MAPDST resist: (A) FE-SEM image of isolated 20, 25, 30, and 35 nm line patterns with 1 : 5 duty cycles; (B) AFM image of 20, 25 and 30 nm lines with L/5S (line/space) patterns.

In previous studies^21^ it was reported the synthesis of the above hybrid copolymers, their EUV exposures and patterning sensitivities based on antimony content, LER/LWR as well as other issues. High resolution isolated and dense 20 nm lines and various complex nano features including waves, boats, pillars, star-elbow *etc.* have been successfully patterned by EUV exposure. The 2.15% MAPDSA–MAPDST resist was capable of patterning 20 nm lines at an exposure dose of 26 mJ cm^−2^ and the results are presented in Fig. 9. As can be seen in Fig. 9, isolated line patterns from 35 to 20 nm were very well resolved. The sensitivity of these newly designed organic–inorganic hybrid resist formulations towards EUV radiation demonstrated that they have better performances as compared to pure MAPDST based organic resists most probably due to the incorporation of hexafluoroantimonate in the polymer back bone. The presence of inorganic SbF_6_^−^, even in low concentration, may lead to a higher etch resistances while maintaining the required processing properties of the resists. In a previous work it was hypothesized that the enhanced sensitivity of the copolymer compared to the base poly-MAPDST homo polymer was due to the higher optical density of Sb relative to carbon.^23^ The present results showed that the SbF_6_^−^ inorganic moiety may control the deposited energy on the resist by gradual homolysis of Sb–F bonds which lost fluorine atoms at a much lower rate that the sulfonium triflate functionality.

Finally, the developed 2.15%-MAPDSA–MAPDST hybrid resist exhibits the following characteristics:^22,23,55^

(i) The resist offered a maximum resolution of 20 nm line features with the maximum sensitivity of 22 mJ cm^−2^ under EUVL (see the image in the ESI†).

(ii) The resist also showed high sensitivity under electron beam (e-beam) and helium ion beam (He^+^ ion) lithography tools for ∼20 nm patterning applications with high sensitivity and low line edge roughness (LER). For example, the calculated sensitivity and LER for 20 nm features exhibiting the resist under He^+^ ion lithography is 7.2 μC cm^−2^ and (1.27 ± 0.31) nm respectively, which are close to the semiconductor roadmap requirements (ITRS-2016).

(iii) The resist exhibited high thermal stability (220 °C), low out gassing properties and compatibility of using industrial standard developer such as TMAH for negative tone patterning.

However, the MAPDSA–MAPDST resist was unable to pattern the sub-10 nm features as the semiconductor industries are particularly looking for efficient ICs production. Therefore, currently our research is focused to improve the resist resolution, particularly for sub-10 nm regime, through structural tuning methods.

## 4 Conclusions

In the present work, a detailed photodynamic study was carried out using SR as an excitation source as well as high surface sensitive analytical tools (NEXAFS and XPS spectroscopy). The investigation clearly showed a fast decomposition rate of the radiation sensitive sulfonium triflate followed with important changes in the ester group. A high rate of defluorination and a loss of sulfonated groups as a result of an increase in the irradiation time for the 2.15% MAPDSA–MAPDST resist thin films were observed. Sulfur L-NEXAFS spectra of the 2.15% MAPDSA–MAPDST resist thin films showed that irradiation at 103.5 eV led to a general decrease in signals, except one signal at about 164.8 eV. This transition was assigned to a –CH_2_–S–CH_2_– functional group, *i.e.*, a Ar–S^+^–(CH_3_)_2_ sulfonium group bonded to the phenyl ring in the case of the 2.15% MAPDSA–MAPDST pristine resist. EUV irradiation of the films showed that the triflate and the ester group are the weakest part of the 2.15% MAPDSA–MAPDST resist.

The detailed HR-XPS results on the energy regions of F 1s and O 1s indicated an important role of the inorganic SbF_6_^−^ moiety during irradiation. It is thought that significant advances would result from synthesizing high EUV absorbance resist materials using heavier atoms, for example, when 13.5 nm photons are used. The obtained results have shown that the inorganic SbF_6_^−^ moiety has a much lower rate of defluorination that the triflate group. Even after 15 min of irradiation, where there was no more sulfonium triflate in the surface region, fluorine linked to antimony was present. The decrease in binding energy of the F–Sb and Sb 3d HR-XPS signals indicated a continuous decrease in the electronegativity of the atoms linked to the antimony during the partial desorption of fluorine when the irradiation time increased.

Finally, the results have shown the complex principles that may govern the photodynamic pathways of photofragmentation of the 2.15% MAPDSA–MAPDST resist: easy decomposition of the radiation sensitive group (sulfonium triflate), polarity switch mechanism due to EUV irradiation with loss of C=O (most likely decarboxylation of ester), transformation of the sulfonium group and the presence of the inorganic SbF_6_^−^ moiety that may control the excess energy deposited on the resist. Further studies of resists incorporating heavy metals are under way with the objective to clarify the actual role of the metals. In addition to surface analysis, fluorescence and outgassing measurements will be carried simultaneously with EUV irradiation. Those results will be published elsewhere. The sensitivity of these newly designed organic–inorganic hybrid resist formulations towards EUVL demonstrated that they have better performances as compared to previous based organic resists.

## Conflicts of interest

There are no conflicts to declare.

## Supplementary Material

RA-008-C7RA12934C-s001

## References

[cit1] https://www.euvlitho.com/2017/2017%20EUVL%20Workshop%20First%20Call%20for%20Papers.pdf

[cit2] Ito H. (2005). Adv. Polym. Sci..

[cit3] MacDonald S. A., Willson C. G., Frechet J. M. J. (1994). Acc. Chem. Res..

[cit4] Ghosh S., Pradeep C. P., Sharma S. K., Reddy P. G., Pal S. P., Gonsalves K. E. (2016). RSC Adv..

[cit5] Bae W. J., Trikeriotis M., Sha J., Schwartz E. L., Rodriguez R., Zimmerman P., Giannelis E. P., Ober C. K. (2010). J. Mater. Chem..

[cit6] Lawrie K. J., Blakey I., Blinco J. P., Cheng H. H., Gronheid R., Jack K. S., Pollentier I., Leeson M. J., Younkin T. R., Whittaker A. K. (2011). J. Mater. Chem..

[cit7] Richard A. L., Laren M. T., Clifford L. H. (2010). J. Vac. Sci. Technol., B..

[cit8] Canalejas-Tejero V., Carrasco S., Navarro-Villoslada F., Garcia Fierro J. L., Capel-Sanchez M. d. C., Moreno-Bondi M. C., Barrios C. A. (2013). J. Mater. Chem. C.

[cit9] Reddy P. G., Mamidi N., Kumar P., Sharma S. K., Ghosh S., Gonsalves K. E., Pradeep C. P. (2016). RSC Adv..

[cit10] Kalyani V., Satyanarayana V. S. V., Singh V., Pradeep C. P., Ghosh S., Sharma S. K., Gonsalves K. E. (2015). Chem.–Eur. J..

[cit11] Cardineau B., Del Re R., Marnell M., Al-Mashat H., Vockenhuber M., Ekinci Y., Sarma C., Freedman D. A., Brainard R. L. (2014). Microelectron. Eng..

[cit12] Sortland M., Hotalen J., Re R. D., Passarelli J., Murphy M., Kulmala T. S., Ekinci Y., Neisser M., Freedman D. A., Brainard R. L. (2015). J. Micro/Nanolithogr., MEMS, MOEMS.

[cit13] Kasahara K., Xu H., Kosma V., Odent J., Giannelis E. P., Ober C. K. (2017). Proc. SPIE.

[cit14] Haitjema J., Zhang Y., Vockenhuber M., Kazazis D., Ekinci Y., Brouwer A. M. (2017). J. Micro/Nanolithogr., MEMS, MOEMS.

[cit15] Satyanarayana V. S. V., Kessler F., Singh V., Scheffer F. R., Weibel D. E., Ghosh S., Gonsalves K. E. (2014). ACS Appl. Mater. Interfaces.

[cit16] Chagas G. R., Satyanarayana V. S. V., Kessler F., Belmonte G. K., Gonsalves K. E., Weibel D. E. (2015). ACS Appl. Mater. Interfaces.

[cit17] Gangnaik A. S., Georgiev Y. M., Holmes J. D. (2017). Chem. Mater..

[cit18] Passarelli J., Murphy M., Del Re R., Sortland M., Dousharm L., Vockenhuber M., Ekinci Y., Neisser M., Freedman D. A., Brainard R. L. (2015). Proc. SPIE.

[cit19] Li L., Chakrabarty S., Spyrou K., Ober C. K., Giannelis E. P. (2015). Chem. Mater..

[cit20] Cameron J. , Thackeray J., Sung J. W., Coley S., Jain V., Ongayi O., Wagner M., LaBeaume P., Kwok A., Valeri D., Hellion M., Icard B., Dal'zotto B., Sourd C. and Pain L., in Extreme Ultraviolet, 2012, vol. 8322

[cit21] Singh V., Satyanarayana V. S. V., Batina N., Reyes I. M., Sharma S. K., Kessler F., Scheffer F. R., Weibel D. E., Ghosh S., Gonsalves K. E. (2014). J. Micro/Nanolithogr., MEMS, MOEMS.

[cit22] Reddy P. G., Thakur N., Lee C. L., Chien S. W., Pradeep C. P., Ghosh S., Tsai K. Y., Gonsalves K. E. (2017). AIP Adv..

[cit23] Reddy P. G., Kumar P., Ghosh S., Pradeep C. P., Sharma S. K., Gonsalves K. E. (2017). Mater. Chem. Front..

[cit24] Ravel B., Newville M. (2005). J. Synchrotron Radiat..

[cit25] Brzhezinskaya M. M., Morilova V. M., Baitinger E. M., Evsyukov S. E., Pesin L. A. (2014). Polym. Degrad. Stab..

[cit26] Unger W. E. S., Lippitz A., Woll C., Heckmann W. (1997). Fresenius. J. Anal. Chem..

[cit27] Okudaira K. K., Yamane H., Ito K., Imamura M., Hasegawa S., Ueno N. (2002). Surf. Rev. Lett..

[cit28] Solomon J. L., Madix R. J., Stohr J. (1991). Surf. Sci..

[cit29] Stohr J., Outka D. A. (1987). Phys. Rev. B: Condens. Matter Mater. Phys..

[cit30] Feng X., Song M.-K., Stolte W. C., Gardenghi D., Zhang D., Sun X., Zhu J., Cairns E. J., Guo J. (2014). Phys. Chem. Chem. Phys..

[cit31] Kaznatcheev K., Dudin P., Lavrentovich O., Hitchcock A. (2007). Phys. Rev. E: Stat., Nonlinear, Soft Matter Phys..

[cit32] Yates B. W., Shinozaki D. M. (1992). J. Mater. Res..

[cit33] Cortes E., Della Vedova C. O., Gerones M., Romano R. M., Erben M. F. (2009). J. Phys. Chem. A.

[cit34] Lud S. Q., Neppl S., Richter G., Bruno P., Gruen D. M., Jordan R., Feulner P., Stutzmann M., Garrido J. A. (2010). Langmuir.

[cit35] Sidelnikova A. L., Andreichuk V. P., Pesin L. A., Evsyukov S. E., Gribov I. V. e., Moskvina N. A. e., Kuznetsov V. L. v. (2014). Polym. Degrad. Stab..

[cit36] Brun S., Guibert G., Meunier C., Guibert E., Keppner H., Mikhailov S. (2011). Nucl. Instrum. Methods Phys. Res., Sect. B.

[cit37] Nansé G., Papirer E., Fioux P., Moguet F., Tressaud A. (1997). Carbon.

[cit38] Bubnova O., Khan Z. U., Malti A., Braun S., Fahlman M., Berggren M., Crispin X. (2011). Nat. Mater..

[cit39] Greczynski G., Kugler T., Keil M., Osikowicz W., Fahlman M., Salaneck W. R. (2001). J. Electron Spectrosc. Relat. Phenom..

[cit40] Feng J., Wen G., Huang W., Kang E.-T., Neoh K. G. (2006). Polym. Degrad. Stab..

[cit41] Birchall T., Connor J. A., Hillier L. H. (1975). J. Chem. Soc., Dalton Trans..

[cit42] Joyner R. W., Lincoln Vogel F. (1981). Synth. Met..

[cit43] Briggs D. and Seach M. P., Practical Surface Analysis. Volume 1. Auger and X-ray Photoelectron Spectroscopy, John Wiley & Sons, Chichester, England, 1996

[cit44] Moulder J. F. , Handbook of X-ray Photoelectron Spectroscopy: A Reference Book of Standard Spectra for Identification and Interpretation of XPS Data, Physical Electronics Division, Perkin-Elmer Corporation, USA, 1992

[cit45] Park J., Yang R. Q., Hoven C. V., Garcia A., Fischer D. A., Nguyen T. Q., Bazan G. C., DeLongchamp D. M. (2008). Adv. Mater..

[cit46] Gamble L. J., Ravel B., Fischer D. A., Castner D. G. (2002). Langmuir.

[cit47] Kasrai M., Brown J. R., Bancroft G. M., Yin Z., Tan K. H. (1996). Int. J. Coal Geol..

[cit48] Sarret G. r., Connan J., Kasrai M., Bancroft G. M., Charrié-Duhaut A., Lemoine S., Adam P., Albrecht P., Eybert-Bérard L. (1999). Geochim. Cosmochim. Acta.

[cit49] Longo L. S., Smith E. F., Licence P. (2016). ACS Sustainable Chem. Eng..

[cit50] Morgan W. E., Stec W. J., Van Wazer J. R. (1973). Inorg. Chem..

[cit51] Sgibnev E. M., Nikonorov N. V., Ignat'ev A. I. (2017). Opt. Spectrosc..

[cit52] Tsukamoto T., Shimada T., Takagi S. (2015). RSC Adv..

[cit53] Liu H. Y., Zhao K. Y., Wang T. T., Deng J. Y., Zeng H. P. (2015). Mater. Sci. Semicond. Process..

[cit54] Sortland M., Hotalen J., Del Re R., Passarelli J., Murphy M., Kulmala T., Brainard R. (2015). J. Micro/Nanolithogr., MEMS, MOEMS.

[cit55] Gonsalves K. E. , Ghosh S., Pradeep C. P., Reddy P. G., Sharma S. K. and Kumar P., Highly Sensitive MAPDSM-MAPDST Based Resists Technology for Next Generation Lithography Applications, Indian Pat. Appl. 201611022219 A, 2016

