# Correction: EUV photofragmentation study of hybrid nonchemically amplified resists containing antimony as an absorption enhancer

**DOI:** 10.1039/c8ra90030b

**Published:** 2018-04-20

**Authors:** Cleverson Alves da Silva Moura, Guilherme Kretzmann Belmonte, Pulikanti Guruprasad Reddy, Kenneth E. Gonsalves, Daniel Eduardo Weibel

**Affiliations:** Department of Chemical Physics, Chemical Institute, UFRGS Porto Alegre 91501-970 RS Brazil danielw@iq.ufrgs.br; School of Basic Sciences, Indian Institute of Technology Mandi Mandi – 175001 Himachal Pradesh India

## Abstract

Correction for ‘EUV photofragmentation study of hybrid nonchemically amplified resists containing antimony as an absorption enhancer’ by Cleverson Alves da Silva Moura *et al.*, *RSC Adv.*, 2018, **8**, 10930–10938.

The authors regret that Kenneth E. Gonsalves’ name was spelled incorrectly in the original article. The correct author names are as presented above.

The Royal Society of Chemistry apologises for these errors and any consequent inconvenience to authors and readers.

## Supplementary Material

