# Supplementary material for: EUV photofragmentation study of hybrid nonchemically amplified resists containing antimony as an absorption enhancer
Source: RSC Adv. 2018 Mar 19;8(20):10930–8. doi: 10.1039/c7ra12934c (PMC9078975; doi:10.1039/c7ra12934c)
Supplement: RA-008-C7RA12934C-s001 [file RA-008-C7RA12934C-s001.pdf]

Electronic Supplementary Material (ESI) for RSC Advances.
This journal is © The Royal Society of Chemistry 2018

# Supporting Information

## EUV Photofragmentation Study of Hybrid Nonchemically Amplified Resists Containing Antimony as Absorption Enhancer

*C. A. S. Moura[a†], G. K. Belmonte[a†], P. G. Reddy[b], K. E. Gonsalves[b] and D. E. Weibel[a*]*

[a]Department of Chemical Physics, Chemical Institute, UFRGS, Porto Alegre, Postal Code: 91501-970, RS, Brazil.
[b]School of Basic Sciences, Indian Institute of Technology Mandi, Mandi – 175001, Himachal Pradesh, India.

*Corresponding author: Tel: +55-5133086204. Fax: +55-5133087304.

E-mail: danielw@iq.ufrgs.br

†These authors contributed equally to this work

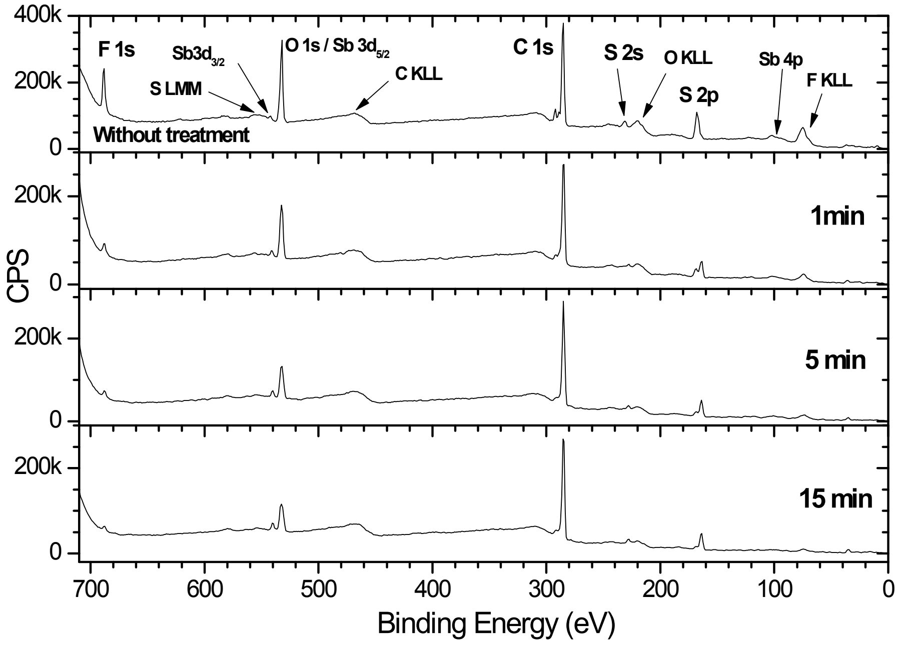


**Figure S1.** XPS wide scan spectra of pristine hybrid non-CAR resist 2.15%-MAPDSA-MAPDST thin film and irradiated at 103.5 eV for 1, 5 and 15 min.

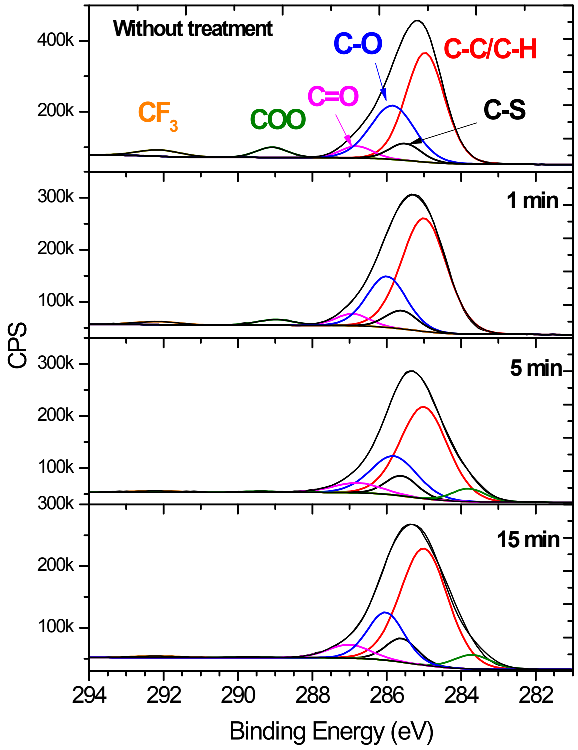


**Figure S2**. High-resolution XPS spectra of the C 1s envelope of pristine hybrid non-CAR resist 2.15%-MAPDSA-MAPDST thin film and irradiated at 103.5 eV for 1, 5 and 15 min.

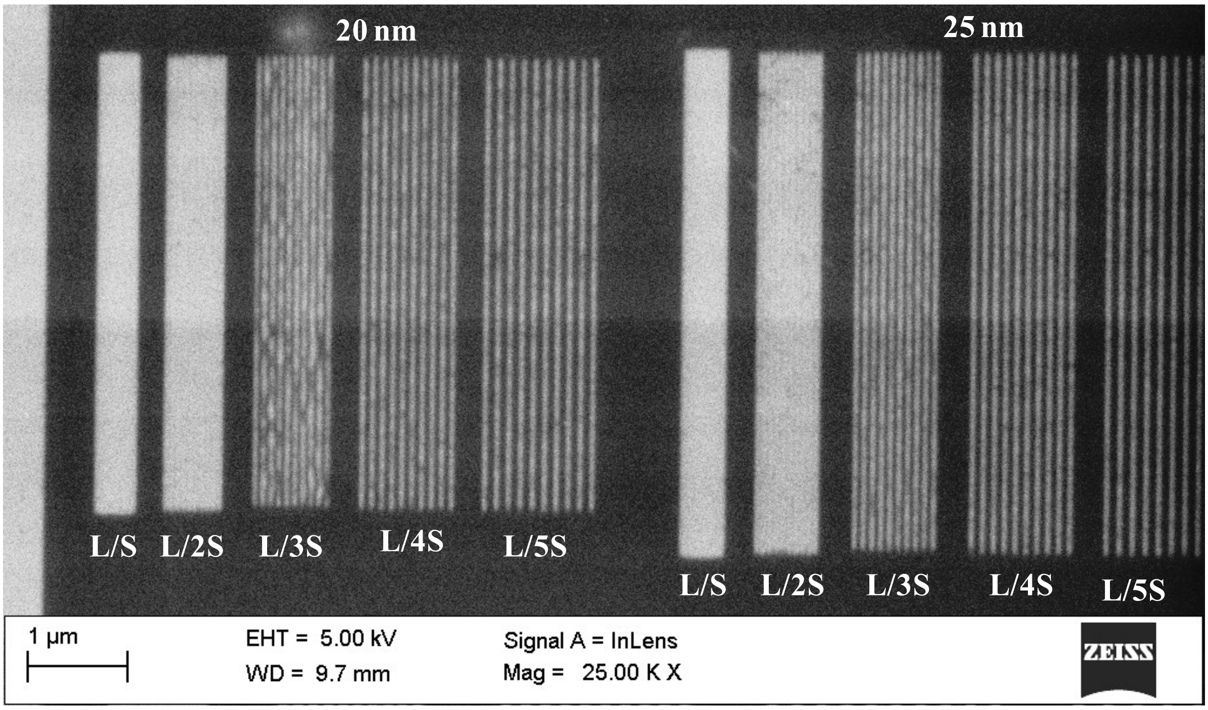


**Figure S3**. SEM image of EUVL patterned 20 and 25 nm (L/S to L/5S) line patterns of 2.15%-MAPDSA-MAPDST resist at the dose ~ 22 mJ/cm2.
